# Transcriptome Analysis Reveals the Molecular Mechanism of the Leaf Yellowing in Allotriploid Cucumber

**DOI:** 10.3390/genes15070825

**Published:** 2024-06-21

**Authors:** Han Wang, Lei Xia, Jinfeng Chen, Chunyan Cheng

**Affiliations:** State Key Laboratory of Crop Genetics & Germplasm Enhancement and Utilization, College of Horticulture, Nanjing Agricultural University, Nanjing 210095, China; 2021104048@stu.njau.edu.cn (H.W.); 2019204026@njau.edu.cn (L.X.); jfchen@njau.edu.cn (J.C.)

**Keywords:** allotriploid, yellowing, transcriptome, chloroplast development, *GLK*

## Abstract

Yellowing leaves are ideal materials for studying the metabolic pathways of photosynthetic pigment chloroplast development, and the mechanism of photosynthetic systems. Here, we obtained a triploid material HCC (2n = 3x = 26), which was derived from hybridization between the artificial tetraploid *Cucumis* × *hytivus* (2n = 4x = 38, HHCC) and the cultivated cucumber *Cucumis sativus* (2n = 2x = 14, CC), and this triploid HCC showed obvious leaf yellowing characteristics. Phenotypic observation results showed that chloroplast development was impaired, the chlorophyll content decreased, and photosynthesis decreased in yellowing HCC leaves. The transcriptome results indicated that HCC-GLK is significantly downregulated in HCC and participates in the regulation of leaf yellowing. GO enrichment analysis revealed that differential genes were enriched in the heme binding and tetrapyrrole binding pathways related to leaf color. KEGG enrichment analysis revealed that differential genes were predominantly enriched in photosynthesis-related pathways. The experimental results of VIGS and yeast hybridization showed that silencing the *GLK* gene can induce leaf yellowing in cucumber plants, and the GLK protein can affect plant chloroplast development by interacting with the CAB3C protein (light-harvesting chlorophyll a/b binding) in the plant chlorophyll synthesis pathway. The current findings have not only enhanced our understanding of the regulatory mechanism of the GLK transcription factor in cucumber but also introduced novel insights and directions for investigating the molecular mechanism underlying polyploid leaf yellowing.

## 1. Introduction

Chlorophyll is one of the key pigments that affect leaf color. Chlorophyll, located in chloroplasts, absorbs and transmits light energy [1]. Chloroplasts play an important role in plant growth and development, serving as the site for photosynthesis as well as supporting the production of hormones and metabolites for plant functioning [2]. Leaf color is an important agronomic trait that affects plant photosynthetic capacity, growth status, ornamental value, and fruit quality [3]. Leaf color mutants are ideal materials for studying photosynthesis mechanisms, chloroplast ultrastructure, chlorophyll biosynthesis pathways, and related gene expression and regulation in plants [4,5].

The molecular mechanisms responsible for the formation of leaf color mutations are very complex, involving many genes and metabolic pathways. Numerous studies have shown that GLK (GOLDEN2-LIKE) is a plant-specific transcription factor which is used to accurately regulate genes involved in photosynthesis, guiding the development and differentiation of chloroplasts and ensuring their normal operation [6,7,8,9,10]. In 1926, Jenkins first identified a maize mutant, *Golden 2* [11]. Subsequently, *GLK* genes have been reported in several species, including moss [12], rice [9], and *Arabidopsis thaliana* [13]. The *glk* mutant in *A. thaliana* leads to the manifestation of a leaf yellowing phenotype [14]. In melon, downregulation of *CmGLK* affects chlorophyll synthesis and chloroplast development [13]. The regulatory role of the *GLK* gene has been demonstrated in rice [9] and tomato [15]. The light-harvesting chlorophyll a/b binding protein is a protein encoded by the *CAB* gene family, which plays an important role in capturing and transmitting light energy [16]. In addition, the expression of *CAB* genes is also related to leaf color. In Arabidopsis and tea trees, it has been found that downregulation of the *lhcb* gene causes yellowing of plant leaves [17,18]. Further research has found that the interaction between light-harvesting chlorophyll a/b binding protein and *GLK* regulates leaf color in peanuts and melon [19,20]. However, the molecular mechanism by which *GLK* specifically regulates chloroplast development in cucumber remains unknown.

Cucumber (*C. sativus* L., 2n = 2x = 14, CC) is one of the most important facility vegetable crops in China. However, the genetic base of cucumber is narrow and lacks intraspecific variation. *Cucumis hystrix* (2n = 2x = 24, HH), a wild species of cucumber, has excellent traits such as resistance to downy mildew, gummy stem blight, and tolerance to low light [21]. Introducing such highly valuable genes from *C. hystrix* into cucumber through interspecific hybridization is an important means for the genetic improvement of cucumber. Previously, Chen and Kirkbride [22] obtained the synthesized tetraploid *C.* × *hytivus* (2n = 4x = 38, referred to hereafter as *C.* × *hytivus*) by interspecific hybridization and doubling techniques. Many triploids have been successfully obtained, derived from the crossing between *C.* × *hytivus* and different cultivated cucumbers [23], which has greatly enriched the genetic diversity of Cucurbitaceae species. Triploids not only have high nutritional value, a unique fruit flavor, and resistance to root-knot nematode [24] but can also be used as excellent intermediate materials for breeding—that is, backcrossing with cultivated cucumber, producing a series of materials containing exogenous high-quality genes such as introgression line and alien addition lines [25]. More significantly, a new triploid material, HCC, was obtained by crossing the synthesized tetraploid *C*. × *hytivus* with *C. sativus*. Compared with other triploids, HCC has yellowing leaves but stronger plant growth and better environmental adaptability. Leaf yellowing in HCC makes it different with the phenotype of the reciprocal triploid CHC, but the mechanism of yellowing is still unknown.

This study assesses the phenotypic features, quantifies the chlorophyll photosynthetic indicators, and examines the chloroplast structures of allotriploid cucumbers (HCC and CHC). The mechanism of leaf yellowing in HCC is explored through transcriptome sequencing (RNA-Seq) and VIGS-validated gene function identification. The results suggest that *GLK* is crucial in the regulatory mechanism of leaf yellowing in HCC. The study provides a basis for understanding the molecular mechanism responsible for leaf yellowing and the cultivation of high-quality triploid germplasm.

## 2. Materials and Methods

### 2.1. Plant Materials

The experimental materials used in this study were *C. sativus* L. var ‘CC3’ (2n = 2x = 14, CC), the tetraploid *C.* × *hytivus* (2n = 4x = 38, HHCC), and the two reciprocal triploids HCC (*C.* × *hytivus* × CC3, 2n = 26) and CHC (CC3 × *C.* × *hytivus*, 2n = 26) by backcrossing *C.* × *hytivus* to *C. sativus* L. (CC3). All plants were preserved and provided by the Cucurbit and Germplasm Enhancement Laboratory of Nanjing Agricultural University. All plants were grown at the Baima Teaching and Research Base of Nanjing Agricultural University. Trait properties were investigated 60 days after transplanting. Each material was assessed using the fourth legitimate leaf, with measurements being taken three times to confirm the consistency of the data.

### 2.2. Measurement of Chlorophyll Contents

The leaves, weighing 0.2 g, were cut into pieces, placed in a 50 mL centrifuge tube, immediately supplemented with 20 mL extract (95% ethanol), and left for 12 h in the dark until the leaf tissue turned white. The light absorption values at 648 nm and 664 nm were determined using UV-Vis (Shimadzu, Kyoto, Japan), and the chlorophyll content was calculated according to the following formula [26]. The absorption value obtained was substituted into the following formula to calculate the content of each pigment. Three biological replicates were set up, with three plants being selected for each biological replicate.

Chla (mg/g) = (13.36 A664 − 5.19 A648) × (20/0.2 × 1000)

Chlb (mg/g) = (27.43 A648 − 8.12 A664) × (20/0.2 × 1000)

### 2.3. Photosynthetic Characteristics and Chlorophyll Fluorescence Parameter Measurement

The photosynthetic characteristics were determined using an Li-6400 portable plant photosynthesis analyzer (LI-COR, Lincoln, NE, USA) from 9 a.m. to 11 a.m. The process was repeated three times for each plant, and the average value of the five plants was determined. An LED red and blue light source was used, the light intensity was set to 1000 µmol·m^−1^, the CO_2_ injection system was set to 400 µmol·mol^−1^, and the gas flow rate was 500 µmol·s^−1^. The parameters included the net photosynthetic rate (Pn), the transpiration rate (Tr), the stomatal conductance (Gs), and the intercellular CO_2_ concentration (Ci). This was repeated three times, with three strains measured each time.

### 2.4. Transmission Electron Microscopy

Small leaf sample pieces (2–3 mm^2^) were excised and promptly immersed in a 2.5% glutaraldehyde fixing solution at 4 °C for 12 h [27]. After fixation, the samples were rinsed with 0.1 mol/L PBS for 30 min (5 repeats). The sample was quickly fixed at 4 °C for 4 h in 0.1 mol/L osmic acid solution. After fixation, it was rinsed with 0.1 mol/L PBS for 30 min and repeated 5 times. Then, gradient dehydration was performed with different concentrations of ethanol, with each stage of dehydration lasting 10–15 min. The sample was then impregnated and wrapped with cyclooxy resin and embedded in an oven at 60 °C for 48 h. Sections were sliced by an ultra-thin microtome (Leica UC7, Wetzlar, Germany) and stained with 2% uranyl acetate and lead citrate. Finally, the samples were observed and photographed using a transmission electron microscope (Hitachi HT7800, Nakanishi, Japan).

### 2.5. RNA Extraction, Library Construction, and Sequencing

Total RNA was extracted from three biological replicates of HCC (yellow leaves) and CHC (green leaves) plants using the RNAprep Plant Mini Kit (Tiangen, Beijing, China) for transcriptomic analysis. RNA integrity and concentration were examined using the Agilent 2100 Bioanalyzer (Santa Clara, CA, USA). A total of 1 µg of RNA per sample was taken as input material for RNA sample preparation. Libraries were generated by the NEBNext® UltraTM RNA Library Prep Kit (Illumina, San Diego, CA, USA). Finally, the constructed cDNA library was sequenced by flow cytometry using the Illumina HiSeq™ 6000 sequencing platform. The sequencing was performed by Guangzhou Kidio Biotechnology Co., LTD (Guangzhou, China).

### 2.6. RNA-Seq Analysis

Raw reads obtained by high-throughput sequencing were filtered to obtain clean reads through Trimmomatic [28]. Trinity software (v2.15.1) was used to assemble all clean data from the transcriptome. The FPKM (fragments per kilobase per million mapped fragments) method was applied to convert the read count number of each gene into an FPKM value representing the gene expression level [29]. Differentially expressed genes (DEGs) were screened with |log2fold change| ≥ 1 and FDR < 0.05 [30]. Gene Ontology (GO) enrichment analysis of the DEGs was performed using Blast2GO software (https://www.blast2go.com/, accessed on 12 June 2024), while Kyoto Encyclopedia of Genes and Genomes (KEGG) enrichment analysis of the DEGs was performed using KOBAS software (https://www.kobas.co.uk/, accessed on 12 June 2024) [31,32].

### 2.7. Validation of DEGs by qRT-PCR

Total RNA was extracted from HCC and CHC leaves using the MiniBEST Plant RNA extraction kit (TaKaRa, Dalian, China), and cDNA was synthesized using the PrimeScript RT reagent Kit with gDNA Eraser (TaKaRa, Dalian, China) according to the instructions provided with the kit. Eight genes were selected for validation, and gene-specific primers were used for qRT-PCR. The primers used in the qRT-PCR experiment are shown in Supplemental Table S2.

### 2.8. HCC-GLK Bioinformatics Analysis

Ex-PASy ProtParam (http://web.expasy.org/protparam/, 15 December 2023) was used to analyze the physicochemical properties of HCC-GLK gene-encoded proteins. BLASTP (https://blast.ncbi.nlm.nih.gov/Blast.cgi, accessed on 15 December 2023) was used for homologous sequence searching. As for sequence alignment and phylogenetic tree construction, the MUSCLE method in MEGA-X and the neighbor joining method (NJ) were applied, respectively [33].

### 2.9. Subcellular Localization of HCC-GLK Protein

For subcellular localization, both HCC-GLK and HCC-GLK:GFP fusion constructs were established. The primers used in the experiments are shown in Supplementary Table S2. The empty vector and the constructed vector were transformed into agrobacteria and then transiently transferred into tobacco leaves through the syringe infiltration method. After dark treatment for 12 h, the tobacco plants were grown under normal conditions for 2 days. After three days of incubation, photos were taken using a laser scanning confocal microscope 900 (Zeiss, Shanghai, China).

### 2.10. VIGS in Cucumber Plants

Cucumber VIGS vector construction was performed using the method described by Liu et al. [34]. The primers used in the VIGS vector construction are shown in Supplementary Table S2. Cucumber seeds were soaked in warm water at around 50 °C and then germinated in a constant temperature incubator at 28 °C. The seeds were sown after germination and placed in a light culture room under a 16 h/8 h cycle at 28 °C with a light intensity of 15,000 Lux. When the cucumber seedlings reached the two true leaf stage, the infective solution was injected into the back of the cucumber cotyledons with a 1 mL syringe.

### 2.11. Yeast Two-Hybrid

**Figure 1:**
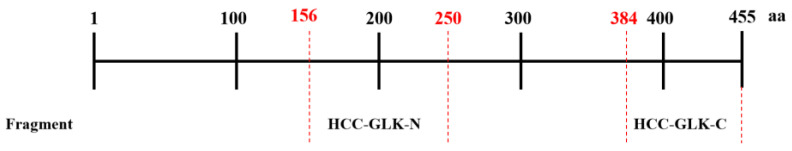
Schematic diagram of HCC-GLK full-length and truncated fragment proteins. The truncation positions are shown with red dashed lines.

The protein domain was predicted based on the mRNA of *HCC-GLK* genes. Then, according to the predicted results, the full-length fragments were truncated without destroying the key structural domains. GLK-N contains the NLS, DBD, and AGREE domains, while HCC-C contains the GCT domain (Figure 1). The short fragments were homologous recombined with the yeast expression vector PGBKT7 (BD). The recombinant vectors of each short fragment separately were transferred into the yeast receptive strain AH109 and applied to SD-Trp, SD-Leu-His, and SD-Leu-His-X-α-gal, and validation of self-activation and toxic growth detection were performed in three types of culture media. Among them, empty pGBKT7 (BD) was the negative control.

Truncated HCC-N (NLS, DBD, and AREAEEA), HCC-C (GCT) domain sequences, and candidate proteins (CAB3C and CAB40) of HCC-GLK were recombined into pGBKT7 (BD) and pGADT7 (AD) vectors and transferred into the yeast strain AH109. It was applied to different amino acid deficiency media, SD-Trp-Leu, SD-Trp-Leu-X-α-gal, SD-Trp-Leu-His-Ade, and SD-Trp-Leu-His-Ade-X-α-gal. The interaction was further determined by yeast growth status and blue staining. Empty AD and empty BD combined were negative controls, while BD-53 and AD-T were positive controls.

## 3. Results

### 3.1. Phenotypic Characteristics and Chlorophyll Content Measurement

**Figure 2:**
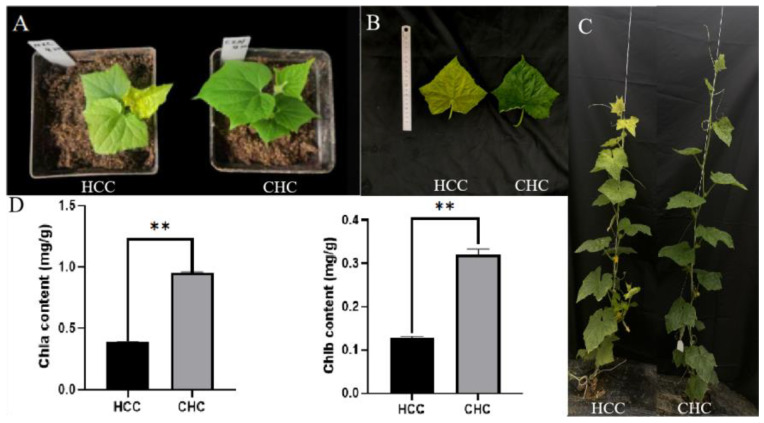
Phenotype and chlorophyll content of the allotriploids. (**A**) Phenotype of HCC and CHC at the seedling stage. (**B**) The fourth true leaves of HCC and CHC. (**C**) Phenotype of HCC and CHC at the outcome stage. (**D**) Chlorophyll content of HCC and CHC. ** *p* < 0.01.

Of the two reciprocal allotriploids (HCC and CHC) we obtained, HCC showed an unusual yellowing leaf phenotype as compared to the reciprocal triploid CHC. The chlorophyll measurements showed (Figure 2D) that the chlorophyll a and chlorophyll b contents of HCC were significantly lower than those of CHC. These results illustrate that the leaf yellowing of HCC plants is directly linked to the lower chlorophyll content. We measured the photosynthetic parameters of HCC and CHC leaves (Figure S2). Compared with CHC, the net photosynthetic rate (Pn), stomatal conductance (Gs), and transpiration rate (Tr) of HCC were significantly reduced, indicating that the photosystem was destroyed and the photosynthetic rate was decreased in the yellowing triploid (HCC).

**Figure 3:**
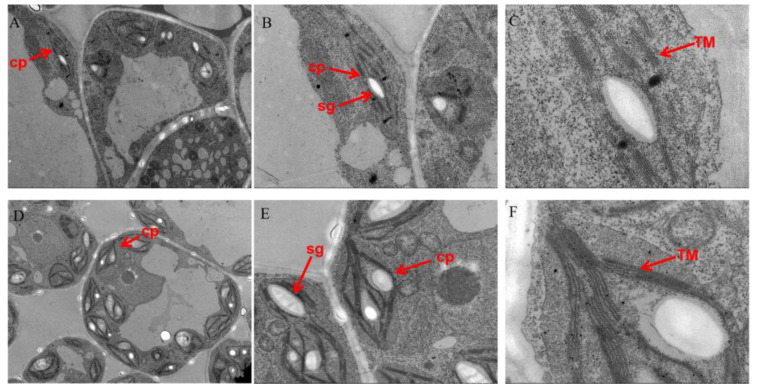
Chloroplast ultrastructure of triploids in *Cucumis*. (**A**–**C**) Chloroplast structure abnormalities in HCC. (**D**–**F**) Chloroplast structure in CHC, which is well developed. Note: sg, starch granule; cp, chloroplast; TM, thylakoid lamellae.

Furthermore, the chloroplast structure of yellow HCC leaves was also examined. As shown in Figure 3, the chloroplast structure of the yellow leaves in HCC was different as most of the basal lamellae were disorganized, exhibiting the presence of large gaps (Figure 3A–C). In contrast, CHC chloroplasts had a complete cell membrane structure, which was mostly pike-shaped or long ellipsoid, with more thylakoid lamellae (TM), a larger number of basal lamellae, and a clearly visible membrane structure of the stromal taxon connecting each basal lamella, with mature starch granules (sg). The results indicate that the chloroplast structure of the yellow leaves in HCC is mutated.

### 3.2. DEGs Analysis and qRT-PCR Verification

Transcriptome sequencing was performed on allotriploid leaves, and the valid data statistics are shown in Table 1. A total of 254,351,774 raw reads were obtained, and 252,683,880 sequences were filtered by removing low-quality raw reads and reads with splices. From Table 1, it can be seen that the percentage of bases with a Phred value greater than 20 is greater than 97%, the percentage of bases with a Phred value greater than 30 is greater than 93% overall, and the sum of the number of bases G and C accounts for a percentage of the total number of bases ranging from 43% to 44%, which indicates that the sequencing quality of the six samples is high enough for subsequent biological analyses. 

In the HCC and CHC samples, 55,284 genes were detected. With FC ≥ 2 and FDR < 0.05 as the screening threshold, 2090 differentially expressed genes (DEGs) were obtained, including 1092 upregulated genes and 998 downregulated genes. Gene expression is represented by volcanic diagrams (Figure S3B). Additionally, qRT-PCR analysis was used to verify the validity of the transcriptomic data (Figure S4). 

### 3.3. GO, KEGG, and TF Analysis of DEGs

**Figure 4:**
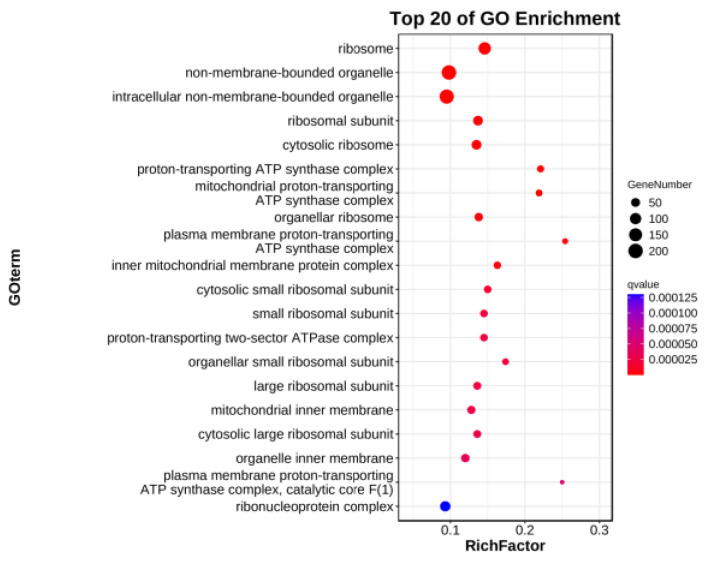
The GO enrichment analysis of differentially expressed genes. The ordinate is the main GO classification categories, and the abscissa is the enrichment factor.

**Figure 5:**
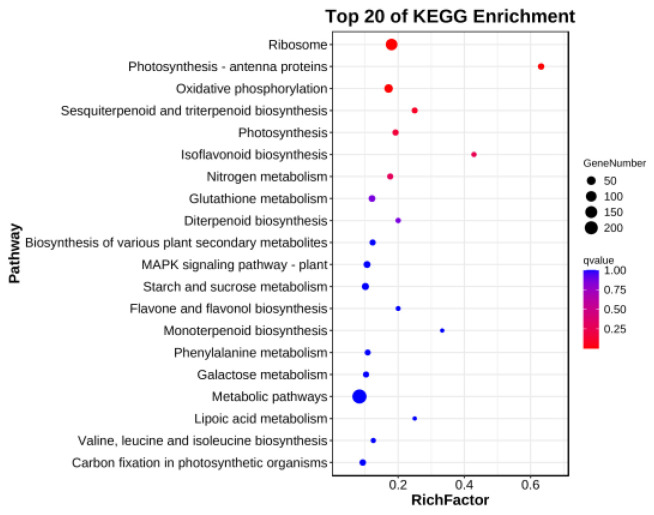
The KEGG enrichment analysis of differentially expressed genes. The ordinate is the main KEGG classification categories, and the abscissa is the enrichment factor.

Differentially expressed genes were subjected to GO analysis. Further enrichment analysis showed that these differential genes were significantly enriched in structural constituents of ribosomes, structural molecular activities, heme binding, and other pathways (Figure 4). Among those related to leaf color are heme binding and the tetrapyrone combination pathway. Thus, it could be inferred that leaf yellowing was related to all these factors. KEGG analysis (Figure 5) revealed that the predominant processes are involved in ribosomes, photosynthesis antenna proteins, etc. The pathway with the highest enrichment factor was that for photosynthesis antenna proteins (with a value of 0.632), in which 12 genes were annotated. These results indicate that the production of yellowed leaves in HCC is closely related to photosynthesis.

**Figure 6:**
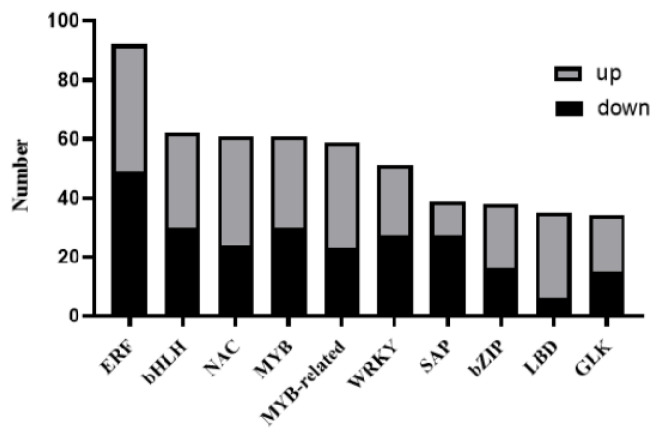
Family classification of transcription factors of DEGs. Note: ERF, ethylene responsive factor; bHLH, basic helix-loop-helix; NAC, NAM, ATAF1/2, and CUC1/2; MYB, v-myb avian myeloblastosis viral oncogene homolog; WRKY, WRKYGOK; bZIP, basic leucine zipper; LBD, lateral organ boundaries domain; GLK, GOLDEN2-LIKE.

Transcription factors (TFs), also known as trans-acting factors, play a key role in the regulation of gene expression, enhancing or inhibiting gene transcription. As can be seen from Figure 6, the maximum number of DEGs encoding ERF transcription factors is 92, while the numbers of genes encoding bHLH and NAC transcription factors are 62 and 61, respectively. Among them, GLK is involved in plant chloroplast development, which is essential for the formation of plant leaf color.

### 3.4. GLK Amino Acid Sequence and Phylogenetic Tree Analysis

**Figure 7:**
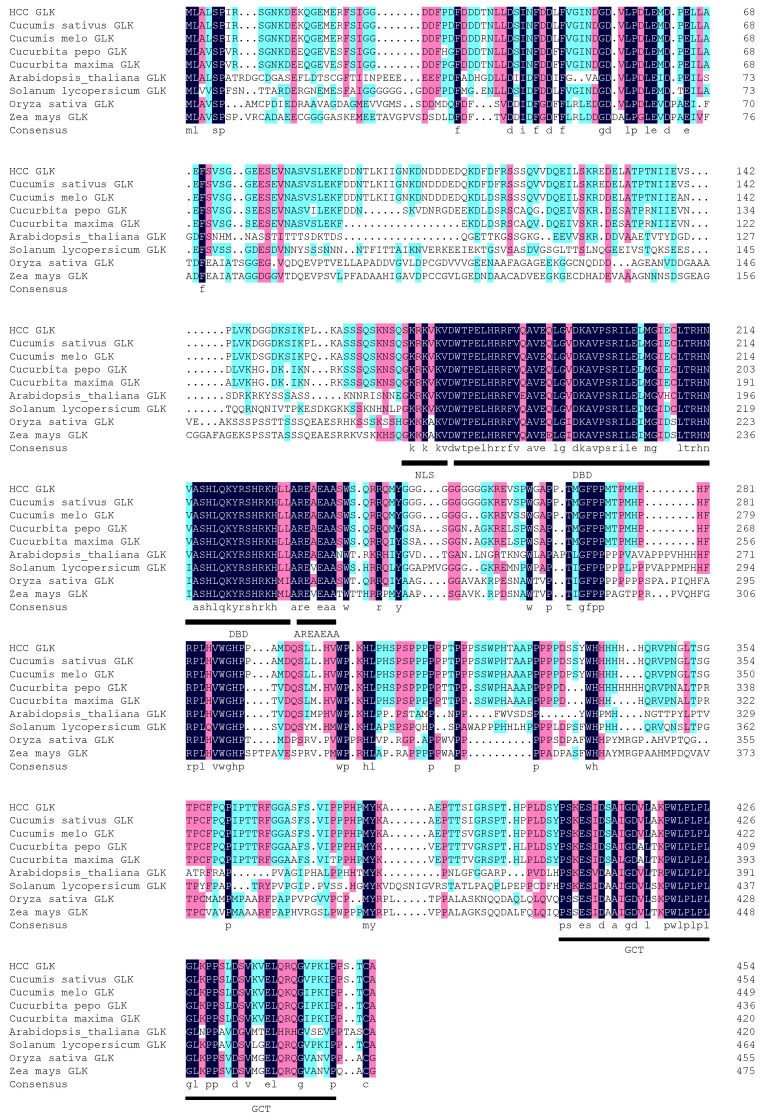
Sequence alignment and phylogenetic tree of *HCC-GLK* and other species. Note: NLS, nuclear localization sequence; DBD, DNA-binding domain; GCT, GOLDEN-2 C-terminal.

The homologous sequences of melon, pumpkin, cantaloupe, Arabidopsis, tomato, rice, and corn were retrieved from the NCBI database, and then an alignment analysis of the homologous amino acid sequences was performed. The results show that there is a certain degree of variation in the GLK amino acid sequence among different species, but the key structural domains (DBD, AREAEAA, and GCT) are relatively conserved (Figure 7). Through comparison, it was found that HCC-GLK and CsGLK had the highest homologous sequence similarity.

**Figure 8:**
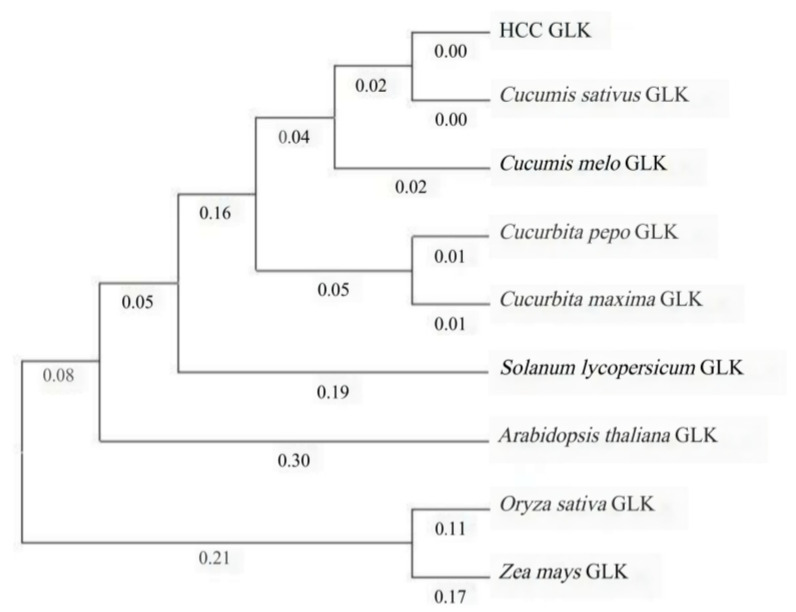
Phylogenetic tree analysis of HCC-GLK based on AA sequences.

Homologous protein sequences of HCC-GLK in different species were compiled into text documents and then imported into MEGA 7.0 to construct a biological evolutionary tree to further study the relationship between different species. The phylogenetic tree was constructed, and it was found that HCC-GLK has the closest genetic relationship with cucumber, followed by melon (Figure 8). Conversely, the most distant genetic relationship was observed with corn and rice.

### 3.5. Subcellular Localization of HCC-GLK

**Figure 9:**
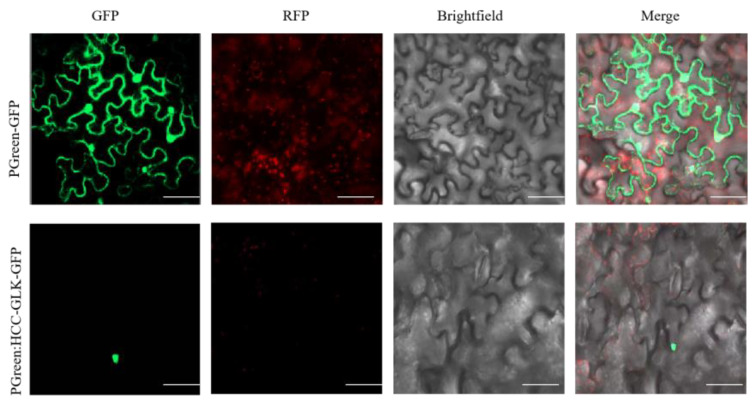
Subcellular localization of HCC-GLK. Bars = 50 μm.

Based on the protein sequence encoded by the *HCC-GLK* gene, it was predicted that the *HCC-GLK* gene encodes a nucleus protein. In order to confirm this prediction, this study used *HCC-GLK*:GFP transiently transferred into tobacco leaves to test the subcellular localization of green fluorescence signals. As predicted, both the GFP signal and nuclear dye DAPI signal were found colocalized in the nucleus, indicating the localization of HCC-GLK protein in the nucleus (Figure 9).

### 3.6. Functional Analysis of HCC-GLK

**Figure 10:**
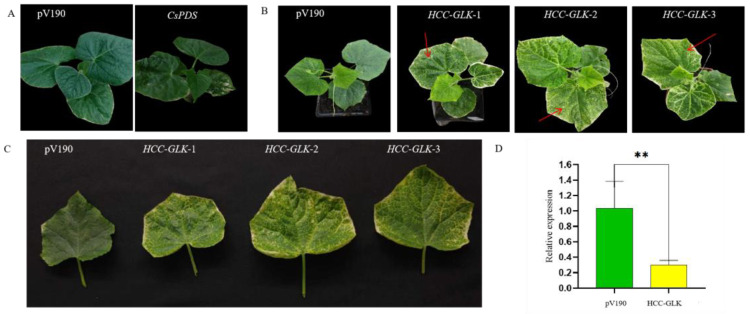
VIGS validation and qRT-PCR analysis. (**A**) Phenotypes of plants silenced by *PDS* gene through VIGS. (**B**) Phenotypes of plants silenced by the *HCC-GLK* gene through VIGS. (**C**) Leaf yellowing in plants silenced by the *HCC-GLK* gene through VIGS. (**D**) Quantitative RT-PCR analysis of *HCC-GLK*, *Pv190:GLK*-silenced plants and Pv190 plants. ** *p* < 0.01.

To verify the function of GLK, virus-induced gene silencing (VIGS) experiments were performed. Firstly, the feasibility of the VIGS experiment was demonstrated by using PDS gene silencing as a positive control, in which the albino phenotype was found in the pV190:PDS positive control. Subsequently, it was observed that the pV190:GLK leaf blades exhibited a significant yellowing effect after injection with pV190:GLK infiltration solution compared with the control pV190 plants,. These observations serve as conclusive evidence that the presence of GLK affects the leaf color of the plants (Figure 10). Furthermore, by qRT-PCR analysis, GLK was also found to affect the expression of genes related to chlorophyll synthesis in silenced plants (Supplementary Figure S2). These results indicate that the transcription factor GLK plays a major role in regulating leaf color yellowing in HCC.

### 3.7. Interplay Analysis in the Yeast Two-Hybrid System

To investigate whether HCC-GLK has transcriptional activation activity, the full-length HCC-GLK protein sequence was inserted into the PGBKT7 vector, and the self-activation of each protein sequence was verified by using the yeast two-hybrid system. The results showed that the full-length HCC-GLK protein transformants grew normally in SD-1, SD-2, and SD-2-X-α-gal media and appeared blue in X-α-gal, indicative of self-activation of the full-length HCC-GLK protein, but were not toxic to yeast cells (Figure S5). In this study, the constructed GLK-N and GLK-C structural protein sequences were incorporated into the PGBKT7 vector, which again verified the self-activation and ensured that the important conserved domain of the HCC-GLK protein was not disrupted. The results showed that GLK-N and GLK-C could grow normally in SD-1 medium but could not grow in SD-2 medium and could not show blue in SD-2-X-α-gal medium, indicating that different truncated proteins had no self-activation and were not toxic to yeast cells (Figure S5).

This study assumed that there may exist some protein-level interactions between the HCC-GLK protein and light-harvesting chlorophyll a/b binding proteins. To accomplish this, light-harvesting chlorophyll a/b binding proteins (CAB3C and CAB40) were inserted into PGADT7 and PGBKT7 vectors, respectively. HCC-GLK-N-BD+CAB3C-AD, HCC-GLK-N-BD+CAB40-AD, HCC-GLK-C-BD+CAB3C-AD, and HCC-GLK-C-BD+CAB40-AD were selected as the experimental groups, and BD+AD and AD-T+BD-53 were used as the control groups. The empty combination was used as the negative control group for yeast two-hybrid point-to-point validation. 

**Figure 11:**
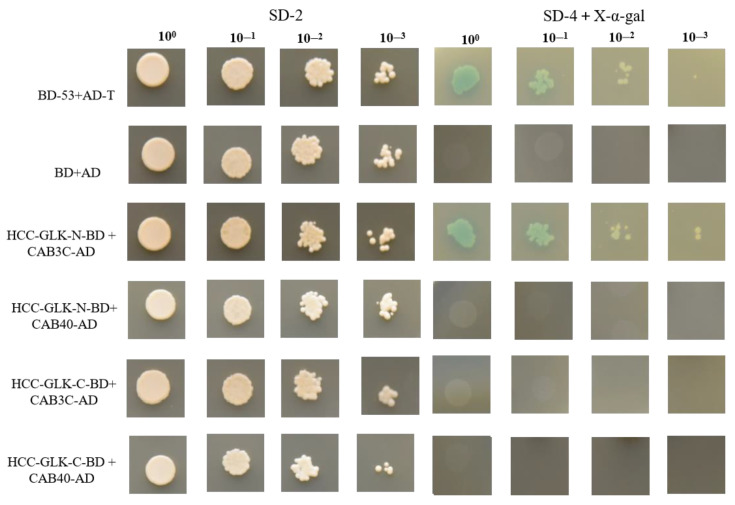
Yeast two-hybrid (Y2H) system of HCC-N-GLK and HCC-C-GLK with CAB40 and CAB3C. SD-2, SD medium lacking Leu and Trp; SD-4+X-α-gal, SD medium lacking Ade, His, Leu, and Trp, plus X-α-gal; BD-53+AD-T and AD+BD used as positive and negative controls, respectively.

The results showed that no growth or blue staining was observed in SD-2 and SD-4+X-α-gal media, which were the negative controls, while the experimental group HCC-GLK-N-BD+CAB3C-AD and the positive control group AD-T+BD-53 showed significant blue staining and normal growth on all media (Figure 11). These results indicate that there was a strong physical interaction between HCC-GLK-N and CAB3C at the protein level.

## 4. Discussion

In this study, chlorophyll content determination and transmission electron microscopy observation showed that HCC leaf yellowing might be related to a reduction in chlorophyll synthesis and impaired chloroplast development. Moreover, through transcriptome analysis, we found that the transcription factor GLK plays an important role in the occurrence of HCC leaf yellowing by regulating chlorophyll synthesis and chloroplast development. These results could provide a basis for analyzing the molecular regulatory mechanism of yellow leaves in polyploids.

Changes in plant leaf color are often due to changes in chlorophyll content. In this study, we found that the chlorophyll content (chlorophyll a and chlorophyll b) of yellow HCC leaves was significantly lower than that of CHC leaves. Most leaf color mutants have varying levels of structural damage in chloroplasts, and differences in the number, shape, and size of chloroplasts lead to differences in leaf color [35]. The ultrastructures of HCC chloroplasts were observed by transmission electron microscopy, which showed that they were severely defective. Further, the number of chloroplasts in HCC leaves was lower than that in CHC leaves. In contrast, the chloroplasts of CHC were structurally complete, clearly visible, and well organized. These results suggest that the yellow leaf phenotype may be caused by some disorganized chloroplast development, which is similar to results in Arabidopsis, rice, and cabbage [36,37,38]. Leaves are important organs for photosynthesis, and changes in the chlorophyll content and chloroplast structure in leaves usually lead to changes in photosynthetic efficiency [39,40,41]. In this study, the net photosynthetic rate of HCC was significantly lower than that of CHC, which was presumably due to the low chlorophyll content and poorly developed chloroplast structure of HCC.

With the rapid development of modern molecular biology technology, transcriptome sequencing (RNA-seq) has been widely used in biological research [42]. In this study, the transcriptome of allotriploid leaves (HCC and CHC) was sequenced, analyzed, and annotated. A total of 2090 DEGs, including 998 upregulated genes and 1092 downregulated genes, were found in the yellow leaves of the triploid HCC. KEGG enrichment analysis showed that the DEGs were enriched in the pathways of chlorophyll synthesis and photosynthesis. Chlorophyll a and chlorophyll b are mainly distributed in the cystoid membrane of chloroplasts and participate in the absorption, transfer, and conversion of light energy in photosynthesis [43]. In this study, we found that many genes related to the photosynthesis-antennal protein pathway were downregulated in the yellowed leaves. Therefore, we speculated that the impaired function of PSI- and PSII-related proteins was the main reason for the decreased photosynthetic performance of yellowed HCC seedlings, which is consistent with the results in *Arabidopsis* [44,45].

In this study, the transcriptome analysis showed that the expression of *HCC-GLK* was significantly downregulated, which may be related to the occurrence of yellowing of HCC leaves. Furthermore, VIGS validation of the *HCC-GLK* gene revealed that downregulation of *HCC-GLK* expression did lead to yellowing in plant leaves. Studies have shown that *AhGLK* is associated with leaf yellowing and activates the expression of the *AhCAB* gene in peanuts [20]. In addition, in melon, the CmGLK protein was found to interact with light-harvesting chlorophyll a/b binding protein, thereby affecting chloroplast development [19]. In this study, the downregulated genes (*CAB3C* and *CAB40*) among genes coding for light-harvesting chlorophyll a/b binding genes were selected to study the GLK regulatory mechanism. Yeast two-hybrid experiments further verified the interaction between the HCC-GLK protein and the light-harvesting chlorophyll a/b binding protein CAB3C, and it was speculated that HCC-GLK and CAB3C jointly regulate allotriploid leaf color, which provides a reference to study the regulatory mechanism of GLK in chloroplast development.

## 5. Conclusions

In this study, physiological characterization of yellowing HCC leaf material revealed that HCC has a lower chlorophyll content, damaged chloroplasts, and reduced photosynthesis, which greatly limits its breeding applications. The transcriptome analysis showed that the yellow leaf phenotype of HCC was possibly caused by the downregulation of the expression levels of *HCC-GLK*. VIGS further confirmed that downregulation of *HCC-GLK* could lead to leaf yellowing. In addition, it was found through yeast two-hybrid experiments that there is an interaction relationship between *HCC-GLK* and *CAB3C*. In conclusion, the results not only provide a theoretical basis for the study of the molecular mechanism of leaf yellowing in polyploid plants but also provide a certain reference for the breeding of excellent triploid germplasm.

## Figures and Tables

**Table 1:** Summary of the sequencing data quality of the RNA-seq.

Sample	Raw Reads	Clean Reads	Clean Bases (bp)	Q20/%	Q30/%	GC/%
HCC-1	50,033,990	49,693,752	7,386,193,583	97.59	93.23	43.43
HCC-2	41,896,320	41,616,444	6,174,711,634	97.25	92.34	43.55
HCC-3	40,254,264	39,981,506	5,938,102,532	97.59	93.01	43.31
CHC-1	45,949,880	45,641,454	6,775,572,126	97.94	94.00	43.86
CHC-2	38,635,066	38,380,724	5,684,103,091	97.76	93.62	43.72
CHC-3	37,582,254	37,370,000	5,537,910,503	97.86	93.59	43.66

## Data Availability

The original contributions presented in the study are included in the article/Supplementary Material, further inquiries can be directed to the corresponding author.

## Supplementary Materials

The following supporting information can be downloaded at: https://www.mdpi.com/article/10.3390/genes15070825/s1, Figure S1: Genetic pedigree of triploids in *Cucumis*; Figure S2: Photosynthetic parameters of allotriploid cucumber; Figure S3: Differential expression gene map; Figure S4: Verification of chlorophyll-related gene expression; Figure S5: Transcriptional activation assay in yeast; Table S1: Assembly quality statistics; Table S2: The primers list used for this study; Table S3: Related gene expression; Table S4: Coding sequence (CDS) and protein sequence of related genes; Table S5: Information on *GLKs* genes in reported species.
