# Supplementary material for: Transcriptome Analysis Reveals the Molecular Mechanism of the Leaf Yellowing in Allotriploid Cucumber
Source: Genes (Basel). 2024 Jun 21;15(7):825. doi: 10.3390/genes15070825 (PMC11275418; doi:10.3390/genes15070825)
Supplement: Supplementary file 1 [file genes-15-00825-s001.zip › genes-3006265-supplementary.pdf]

Transcriptome analysis reveal the molecular mechanism of the leaf yellowing in allotriploid cucumber

Han Wang[1†], Lei Xia[1†], Jinfeng Chen[1] and Chunyan Cheng[1*]

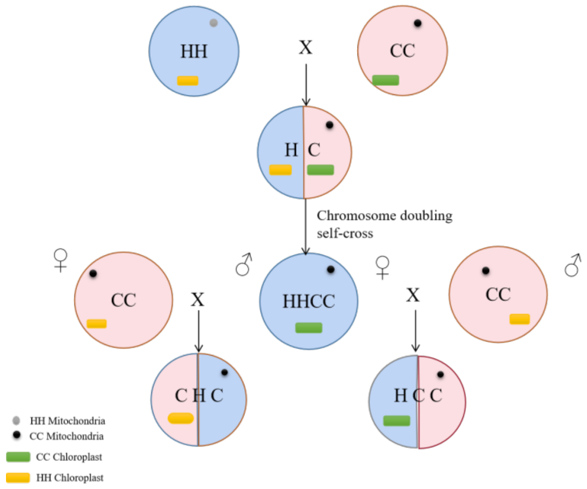

Supplementary Figure S1. Genetic pedigree of triploids in *Cucumis.* HH stands for *C. hystrix*, CC stands for *C. sativus* L. var 'CC3', HC stands for interspecies hybrid F1, HHCC stands for tetraploid *C.* × *hytivus*, and CHC and HCC stand for triploids in *Cucumis.*

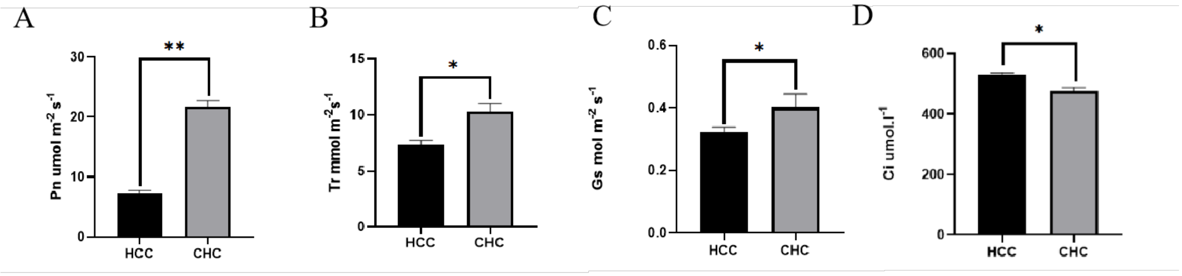

Supplementary Figure S2. Photosynthetic parameters of allotriploid cucumber.

A:Pn; B:Tr; C:Gs; D:Ci

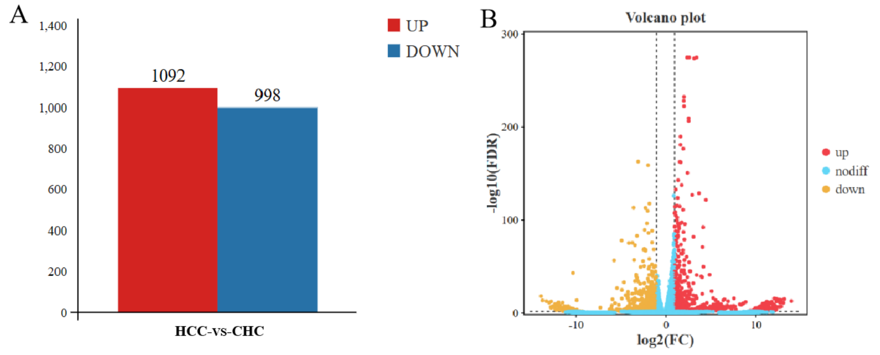

Supplementary Figure S3. Differential expression gene map.

A: Diaglots of differential gene numbers; B:Volcano map of the differential genes

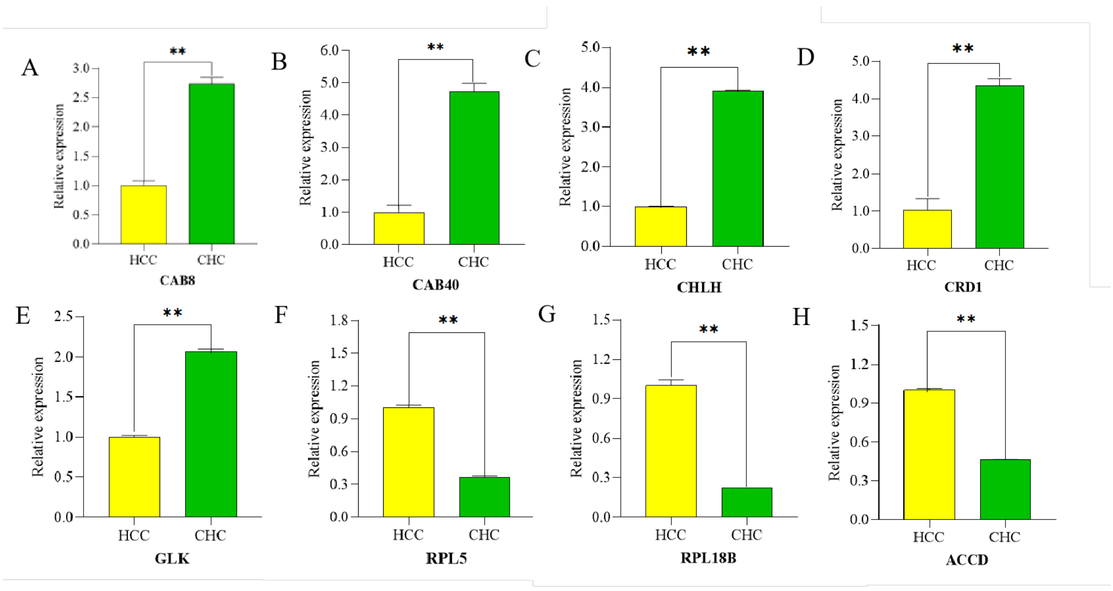

Supplementary Figure S4. Verification of chlorophyll related gene expression.

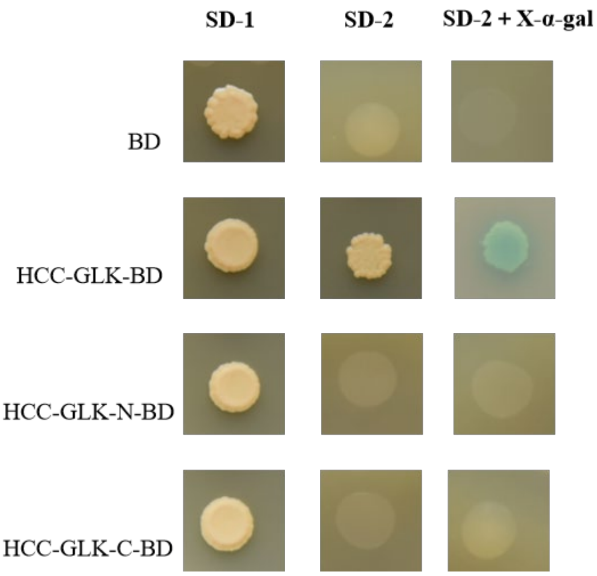

Supplementary Figure S5. Transcriptional activation assay in yeast.
SD-1, SD media lacking Leu; SD-2+X-α-gal, SD media lacking His and Ade plus X-α-gal.
BD was used as the negative controls, respectively.

Supplementary Table S1 Assembly quality statistics

Genes Num	GC percentage	N50 number	N50 length	Max length	Min length	Average length	Total assembled bases
55,284	38.8987	8,452	2,210	21,937	201	1,074	59,390,584

Supplementary Table S2 The primers list used for this study.

Gene	Forward primer	Reverse primer
actin	ATTGTTCTCAGTGGTGGTTCTAC	ATTGTTCTCAGTGGTGGTTCTAC
CAB8	CAAGATGGTTGGCATACGG	TAGTTGTAGGTCCCTGCTGGT
CAB40	GAGCCACCATCCTTCCTTAC	CTTCACCAAACTTCACACCATT
CHLH	GACCGATGTCAGCCATTACTT	GCATCCAGTCTCACCGTCTC
CRD1	AGGATGAGAACAGGCACGG	GCAGAAGAATCGAGACCACAA
GLK	GATGAGAAACAAGGAGAAATGGAG	TCAGCGAGGAGTTCAGGGTC
RPL5	GCCCTATCTGCCTTGTTGTT	TGGGGTATGTGTTCTTTCTTGA
RPL18B	GCTCCTCTGGGTCATAAAACA	GCCCTCTCAAACTTCCTTCC
ACCD	ATGGCAAAAATATCTTCCGC	GCTGTCACCCCACCAGTAGTA
GLK-PGreen	TACATCTAGAGGATCCCCATGTTGGAGAAAGGCATTATTG	GACCGGCCGGTGGATCCCGTTGTGCCAATTGTGTTTC
GLK-VIGS	AGGACTTTACTTAATGGATCCATGAACCCCTAAAGGCATCTTCTTCT	CTAGACCTATAACTGGATCCTGGCTCCAACTCGCCG

Supplementary Table S3 Related gene expression

Genes	CHC_mean	HCC_mean	log2(fc)
ACCD	8.860666667	25.25266667	1.510948589
CRD1	149.683	59.36333333	-1.33426637
CHLH	215.0796667	83.69066667	-1.361732496
GLK	19.16033333	6.554666667	-1.54752834
RPL5	0.418333333	1.831333333	2.130169546
RPL18B	0.001	6.758	12.72238064
CAB3C	963.032	183.63	-2.390781963
CAB7	1146.094	384.7483333	-1.574738393
CAB8	871.633	291.8786667	-1.578352049
CAB13	1216.708667	293.8946667	-2.049612681
CAB40	3270.249333	613.4623333	-2.414353965

Supplementary Table S4 Coding sequence (CDS) and protein sequence of related genes

Genes	CDS	Protein
GLK	ATGCTTGCCTTGTCACCTATTAGAAGTGGCAACAAAGATGAGAAACAAGGAGAAATGGAG<br>AGGTTTTCGATTGGAGGTGATGATTTTCCCGATTTTGATGATGATACAAATTTGCTTGATAG<br>CATCAACTTTGATGATCTTTTTGTGGGAATCAACGACGGAGATGTGTTGCCTGATTTGGAGA<br>TGGACCCTGAACTCCTCGCTGAATTCTCCGTTAGCGGCGGTGAGGAATCGGAAGTCAACGC<br>ATCAGTTTCTTTAGAAAAATTCGACGACAATACATTAAAGATTATCGGTAATAAAGACAAC<br>GACGATGATGAAGATCAGAAAGATTTTGATTTTAGATCTTCTAGCCAAGTGGTGGATCAAG<br>AGATTTTGAGTAAACGAGAAGATGAATTGGCCACACCAACAAATATTATTGAAGTAAGCC<br>CTTTGGTGAAAGATGGTGGCGATAAAAGTATTAAACCCCTAAAGGCATCTTCTTCTCAATC<br>CAAAAATTCTCAAAGCAAGAGAAAAGTTAAGGTTGATTGGACACCGGAGCTGCACCGGCG<br>GTTTGTACAAGCCGTTGAGCAATTGGGTGTGGATAAGGCAGTTCCTTCTAGAATATTGGAG<br>CTTATGGGAATTGAGTGTCTCACTCGCCATAACGTTGCTAGCCATCTTCAAAAATATAGGTC<br>GCATCGGAAACATTTGTTGGCACGTGAAGCGGAGGCGGCGAGTTGGAGCCAAAGGCGGCA<br>GATGTACGGAGGCGGTGGCGGTGGAGGTGGAGGCGGAAAGAGAGAGGTGAGTCCATGGG<br>GTGCACCGCCCACCATGGGATTTCCGCCCATGACGCCCATGCACCCTCATTTTAGGCCTTTA<br>CACGTGTGGGGTCATCCTCCTGCTATGGATCAATCTCTTTTGCACGTGTGGCCTAAGCATCT<br>TCCTCATTCACCATCTCCTCCGCCACCTCCACCTACTCCTCCGCCATCCTCATGGCCACACA<br>CCGCCGCTCCTCCTCCGCCCCCTGACTCTTCCTACTGGCACCACCACCACCACCACCAACG<br>GGTTCCAAATGGTTTAACCTCAGGAACGCCATGCTTCCCACAGCCAATACCAACCACGAGA<br>TTTGGTGGGGCAAGTTTCTCGGTAATCCCACCGCCTCATCCGATGTACAAAGCAGCTGAGC<br>CCACAACGAGCATAGGGCGTTCTCCCACTCACCCTCCCCTCGACTCTTATCCATCGAAAGA<br>GAGTATTGACTCAGCAATTGGAGATGTGTTAGCGAAGCCATGGCTACCCCTTCCTCTTGGAT<br>TAAAGCCTCCCTCTTTAGACAGTGTTAAAGTTGAACTCCAAAGACAAGGAGTTCCTAAAAT<br>ACCTCCCAGTACTTGTGCTGCTTAA	MLALSPIRSGNKDEKQGEMERFSIGGDDF[illegible]<br>FDDDTNLLDSINFDDLFVGINDGDVLPDL[illegible]<br>DPELLAEFSVSGGEESEVNASVSLEKFDDN<br>KIIGNKDNDDDEDQKDFDFRSSSQVVDQE<br>SKREDELATPTNIIEVSPLVKDGGDKSIKPL<br>SSSQSKNSQSKRKVKVDWTPELHRRFVQA<br>QLGVDKAVPSRILELMGIECLTRHNVASH[illegible]<br>KYRSHRKHLLAREAEAASWSQRRQMYG[illegible]<br>GGGGGGGKREVSPWGAPPTMGFPPMTP[illegible]<br>PHFRPLHVWGHPPAMDQSLLHVWPKHL[illegible]<br>SPSPPPPPPTPPPSSWPHTAAPPPPPDSSYW<br>HHHHHQRVPNGLTSGTPCFPQPIPTTRFG[illegible]<br>SFSVIPPPHPMYKAAEPTTSIGRSPTHPPLD[illegible]<br>PSKESIDSAIGDVLAKPWLPLPLGLKPPSLD<br>KVELQRQGVPKIPPSTCAA

Continued table

Genes	CDS	Protein
CAB3C	ATGGCAGCCATGGCTCTTTCATCTACATTCACAGGAAAAGTTGTTCCTTTGAATGCATTTAC<br>CGAGCTCTCGTCCTCGGTTCGCAGCAACGGTAGAGTCACAATGAGGAAATCTGGAAAGCC<br>AGCAGCTTCCTCAGGCAGCCCATGGTATGGTCCTGACCGTGTCAAGTACCTTGGACCGTTCT<br>CTGGTGAGCCTCCATCTTACCTCAAAGGTGAATTCCCTGGTGACTATGGTTGGGACACTGCA<br>GGTCTATCAGCTGACCCCGAGACATTTGCCAAGAACCGAGAGCTCGAAGTGATTCACAGT<br>AGATGGGCCATGCTTGGGGCTCTAGGATGTGTATTCCCTGAGCTTCTTTCTAGAAATGGAGT<br>CAAGTTTGGTGAAGCAGTTTGGTTCAAGGCTGGTTCACAAATCTTCAGTGAGGGTGGCCTA<br>GACTATCTTGGAAACCCAAGTCTTATTCATGCACAGAGCATTTTGGCAATTTGGGCATCTCA<br>AGTAGTGTTAATGGGTGCTGTTGAAGGTTACCGTATTGCTGGAGGACCACTCGGAGAGATT<br>ACTGATCCCATCTACCCAGGTGGTAGCTTTGATCCCCTTGGGTTGGCTGATGATCCAGAGGC<br>ATTTTCAGAACTTAAGGTGAAAGAACTTAAGAATGGTCGTCTTGCAATGTTTTCCATGTTCG<br>GGTTTTTCGTACAGGCCATTGTAACTGGAAAAGGTCCTTTGGAGAACCTTGCAGACCATCTT<br>GCTGATCCTGTCAACAACAATGCATGGGCTTATGCTACCAACTTTGTACCTGGAAAGTGA	MAAMALSSTFTGKVVPLNAFTELSSSV<br>RSNGRVTMRKSGKPAASSGSPWYGPD<br>RVKYLGPFSGEPPSYLKGEFPGDYGWD<br>TAGLSADPETFAKNRELEVIHSRWAML<br>GALGCVFPELLSRNGVKFGEAVWFKA<br>GSQIFSEGGLDYLGNPSLIHAQSILAIW<br>ASQVVLMGAVEGYRIAGGPLGEITDPIY<br>PGGSFDPLGLADDPEAFSELKVKELKN<br>GRLAMFSMFGFFVQAIVTGKGPLENLA<br>DHLADPVNNNAWAYATNFVPGK

Continued table

Genes	CDS	Protein
CAB40	ATGGCTGCTTCTACAATGGCTCTCTCCTCCCCAACTCTGGCCGGCCAAGCCGTGAAACTTT<br>CCCCCAATGCCCCTGAAATTCAGGGCAATGCAAAATTCACCATGAGGAAGACTGCCAGC<br>AAGTCAGTTTCTTCCGGCAGCCCGTGGTATGGTCCTGACCGTGTCAAGTACTTGGGCCCAT<br>TCTCTGGTGAGCCACCATCCTACCTTACCGGAGAGTTCCCTGGTGACTACGGTTGGGACAC<br>TGCTGGACTTTCAGCTGATCCCGAGACCTTCGCCAAGAACCGTGAGTTGGAAGTGATCCA<br>CTCCAGATGGGCCATGCTTGGAGCTTTGGGGTGTGTTTTCCCTGAGCTTTTGTCTCGTAATG<br>GTGTGAAGTTTGGTGAAGCTGTGTGGTTCAAGGCTGGTTCGCAGATCTTCAGCGAGGGCG<br>GGCTTGACTACTTGGGCAACCCCAGCTTGGTCCACGCTCAGAGCATTTTGGCCATCTGGGC<br>TTGCCAAGTTGTGTTGATGGGTGCCGTCGAGGGTTACCGTATTGCTGGTGGCCCACTCGGA<br>GAGGTGACTGACCCCATCTACCCAGGTGGAAGCTTCGACCCATTGGGTTTGGCTGATGAC<br>CCAGAGGCATTCGCTGAGTTGAAGGTTAAGGAGCTCAAGAATGGAAGGTTAGCTATGTTC<br>TCCATGTTTGGATTCTTTGTTCAGGCTATCGTTACCGGAAAGGGTCCATTGGAGAATTTGG<br>CTGATCACCTTGCTGACCCAGTCAACAACAATGCTTGGGCCTATGCTACAAACTTTGTTCC<br>TGGAAAGTGA	MAASTMALSSPTLAGQAVKLSPNAPEI<br>QGNAKFTMRKTASKSVSSGSPWYGPD<br>RVKYLGPFSGEPPSYLTGEFPGDYGWD<br>TAGLSADPETFAKNRELEVIHSRWAML<br>GALGCVFPELLSRNGVKFGEAVWFKA<br>GSQIFSEGGLDYLGNPSLVHAQSILAIW<br>ACQVVLMGAVEGYRIAGGPLGEVTDPI<br>YPGGSFDPLGLADDPEAFAELKVKELK<br>NGRLAMFSMFGFFVQAIVTGKGPLENL<br>ADHLADPVNNNAWAYATNFVPGK

Supplementary Table S5 Information on *GLKs* genes in reported species

Spieces	Accession
Cucumis sativus	XP_004138790.1
Cucumis melo	XP_008455954.2
Cucurbita pepo	XP_023512096.1
Cucurbita maxima	XP_022986312.1
Arabidopsis thaliana	NP_565476.1
Solanum lycopersicum	NP_001266193.1
Oryza sativa	XP_015644244.1
Zea mays	NP_001105018.1
